# Supplementary material for: Signal transduction interfaces for field-effect transistor-based biosensors
Source: Commun Chem. 2024 Feb 19;7:35. doi: 10.1038/s42004-024-01121-6 (PMC10876964; doi:10.1038/s42004-024-01121-6)
Supplement: Supplementary file 2 — SUPPLEMENTAL MATERIAL [file 42004_2024_1121_MOESM2_ESM.pdf]

Supplementary Information

Signal transduction interfaces for field-effect transistor-based biosensors

Toshiya Sakata*
Department of Materials Engineering, School of Engineering, The University of Tokyo, 7-3-1 Hongo, Bunkyo-ku, Tokyo 113-8656, Japan
*Corresponding author. E-mail: sakata@biofet.t.u-tokyo.ac.jp

Trend of research studies on MIPs

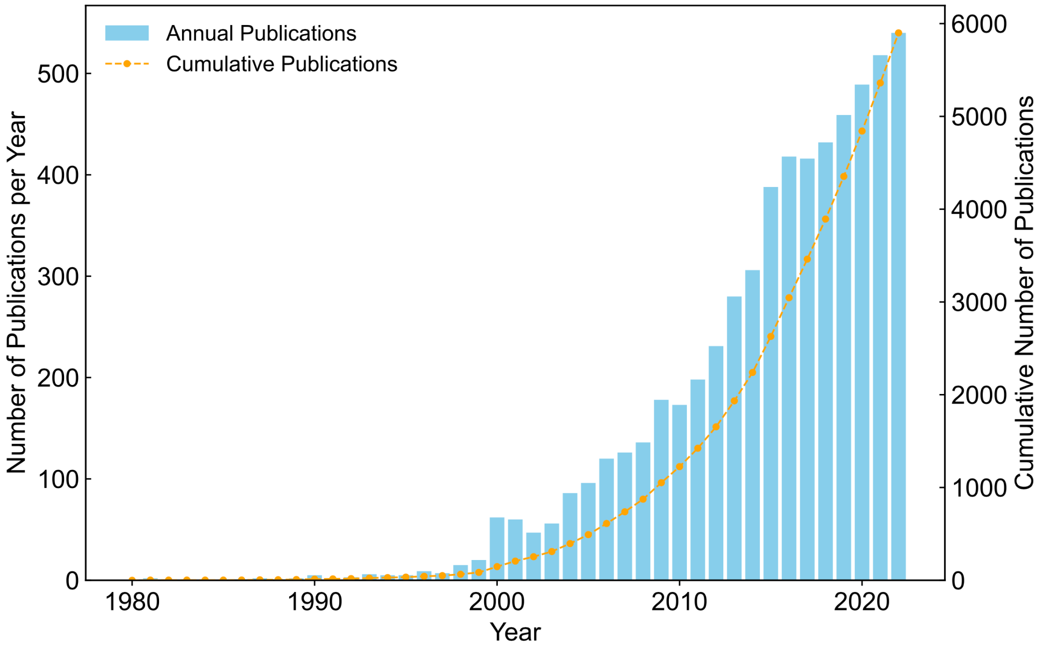

Figure S1. Yearly number and cumulative number of publications on MIPs obtained by a search in PubMed, where "molecularly imprinted polymer" was used as a keyword.

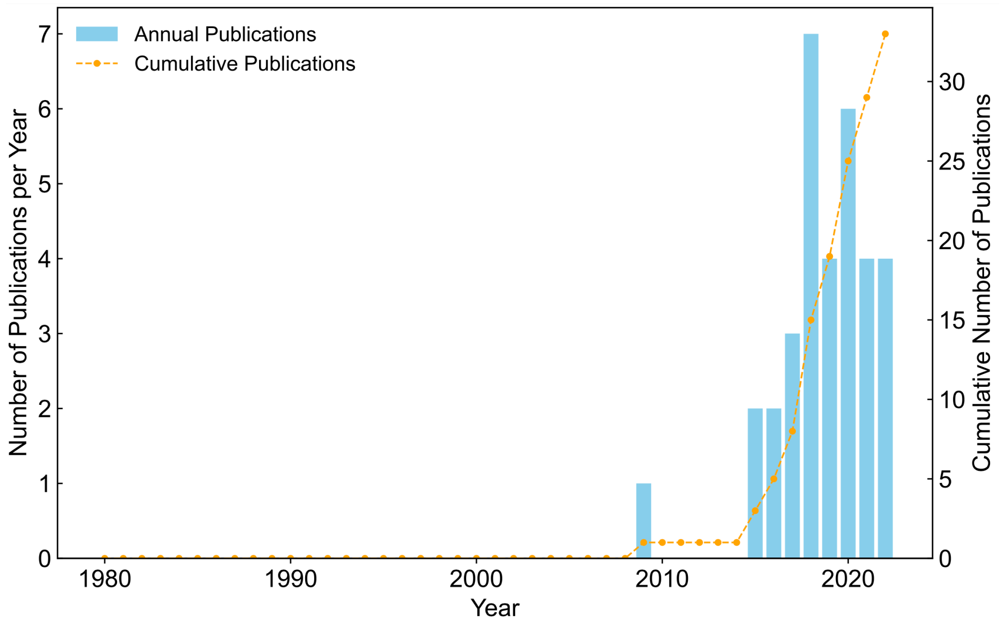

Figure S2. Yearly number and cumulative number of publications on MIP-based FET biosensor obtained by a search in PubMed, where "molecularly imprinted" and (not or) "transistor" were used as keywords.
